# Alcohol Consumption and Risk for Venous Thromboembolism: A Meta-Analysis of Prospective Studies

**DOI:** 10.3389/fnut.2020.00032

**Published:** 2020-04-02

**Authors:** Mengyan Chen, Mingxia Ji, Tiejiang Chen, Xiaofei Hong, Yian Jia

**Affiliations:** ^1^Department of Emergency, Yiwu Central Hospital, Yiwu, China; ^2^Department of Gastroenterology, Yiwu Central Hospital, Yiwu, China

**Keywords:** alcohol drinking, venous thromboembolism, pulmonary embolism, cohort, meta-analysis

## Abstract

The association between alcohol consumption and venous thromboembolism (VTE) risk has been investigated by various observational studies with inconsistent results. We examined this association by performing a meta-analysis of prospective studies. A comprehensive literature search was carried out in the PubMed, EMBASE, and Web of Science from its inception to February 2020. Pooled effect estimates were calculated using a random effect model. Ten prospective studies (14 cohorts) were included in this meta-analysis with a total of 441,128 individuals and 10,221 VTE cases. Overall, the highest consumption of alcohol was not associated with the VTE risk compared with the lowest group [relative risk (RR), 0.96 (95% CI, 0.89–1.04), *P* = 0.293]. No obvious heterogeneity of RRs was observed across these studies (*P* = 0.249 for heterogeneity, *I*^2^ = 18.8%). Egger's and Begg's tests showed no evidence of publication bias (Egger, *P* = 0.443; Begg, *P* = 0.730). In the subgroup analysis by sex, a borderline significant association between alcohol consumption and VTE risk was observed in women [RR, 0.91 (95% CI, 0.82–1.00)]. In the dose–response analysis, we observed a linear decrease in VTE risk with increasing alcohol intake (*P* = 0.634 for nonlinearity). However, the reduced risk was not statistically significant. In conclusion, the results from this meta-analysis suggest that alcohol intake is not related with the risk of VTE. Further large well-designed cohort studies are warranted to investigate a potential protective role of alcohol against VTE in women.

## Introduction

Venous thromboembolism (VTE) includes both pulmonary embolism (PE) and deep vein thrombosis (DVT), with an estimated annual incidence rate of 1–2 events per 1,000 person-years (1). Trauma, cancer, pregnancy, and surgery are the well-established risk factors for provoked VTE (2–4). Approximately 25–40% of incident VTE events are unprovoked (idiopathic) in nature (5, 6), and lifestyle characteristics [e.g., lack of physical activity (7) and smoking (8)] may be important risk factors.

Low to moderate alcohol consumption has been associated with a decreased risk of arterial thrombosis (9). The association between alcohol consumption and VTE risk also has been investigated by various observational studies. Some studies reported a reduced VTE risk associated with higher alcohol intake (10, 11), others reported a U-shaped relationship (12), and yet others reported no association (13–15). Understanding the effects of alcohol consumption on risk of VTE will contribute to early detection, potentially improving the prognosis of patients. To our knowledge, no meta-analysis has been performed on this topic. We therefore performed the present dose–response meta-analysis on alcohol intake and VTE risk by summarizing the results of all eligible prospective studies.

## Methods

### Literature Research

The present meta-analysis was performed in accordance with the Preferred Reporting Items for Systematic Reviews and Meta-Analyses guidelines (16). A comprehensive literature search was carried out in the PubMed, EMBASE, and Web of Science from their inception to February 2020. The following keywords and their combinations were used as search strategy: (“alcohol” or “alcohols” or “alcoholic” or “ethanol” or “wine” or “beer” or “liquor”) and (“VTE” or “thromboembolism” or “venous thrombosis” or “pulmonary embolism”) and (“cohort” or “prospective” or “nested case–control” or “trial”). The search strategy in PubMed is provided in Supplementary Text S1. Similar search strategy was used in EMBASE and Web of Science. The reference lists of retrieved articles and reviews were examined for additional relevant studies. There were no restrictions for language or publication date.

### Inclusion and Exclusion Criteria

Eligibility criteria were reported using the PICOS strategy (Supplementary Table S1). Included studies must meet all the following criteria: (1) cohort or nested case–control study or randomized trial; (2) evaluated the association between alcohol intake and VTE risk; and (3) reported relative risk (RR) and its 95% confidence interval (CI). There were no restrictions on types of VTE, which included fatal or nonfatal, symptomatic or asymptomatic, and provoked or unprovoked VTE. For articles based on same study population, the article reporting the largest sample size or longest follow-up was included. Study selection was independently performed by two reviewers (MC and MJ). Any divergences were resolved by consensus.

### Data Extraction

The following information was recorded from each included study with a predesigned data collection form: first author's surname, study country, study design, study population/source, duration of follow-up, number of cases and sample size, method of exposure assessment, and risk estimates with their 95% CIs. Data extraction was performed by the same two independent reviewers (MC and MJ).

### Assessment of Study Quality

The quality evaluation was performed with an eight-item instrument named Newcastle–Ottawa Scale (NOS, http://www.ohri.ca/programs/clinical_epidemiology/oxford.asp). Each item represents 1 point, except for Comparability (2 points). A study with ≤6 points was considered low quality, and >6 points was considered high quality. The quality assessment was carried out by two independent reviewers (MC and MJ). Any disagreements were resolved through discussion and consensus.

### Statistical Analysis

The summary RR with its 95% CI was calculated using a DerSimonian random effect model that accounted for both within- and between-study heterogeneity (17). Heterogeneity across included studies was assessed using the Cochran chi-square test (the level of significance was set at 0.1) and the *I*^2^ statistic (18). Publication bias was assessed using a visual plot, Begg's test (rank correlation method) (19), and Egger's test (linear regression method) (20). Sensitivity analysis was performed by removing each included study in turn and then recalculating the summary risk estimate. Subgroup analyses were also carried out based on the following factors: geographical region, gender, number of cases, and publication year.

Next, studies that reported at least three levels of alcohol intake and corresponding VTE risk estimates were included in the dose–response analysis. For studies that only provided a range for exposures, the mid-point in each category was assigned to the corresponding RR. When the highest category was open ended, the width of the interval was assumed to be the same as in the closest category. Generalized least squares trend (GLST) regression model (21, 22) was used to explore the potential dose–response relation between alcohol intake and VTE risk. Finally, a potential nonlinear dose–response association was examined by modeling alcohol intake using restricted cubic splines with three knots at percentiles 25, 50, and 75% of the distribution (23). Statistical analyses were performed with STATA 10.0 (StataCorp, College Station, TX) with two-sided *P* values (set at 0.05).

## Results

### Study Selection and Main Characteristics

**Figure 1:**
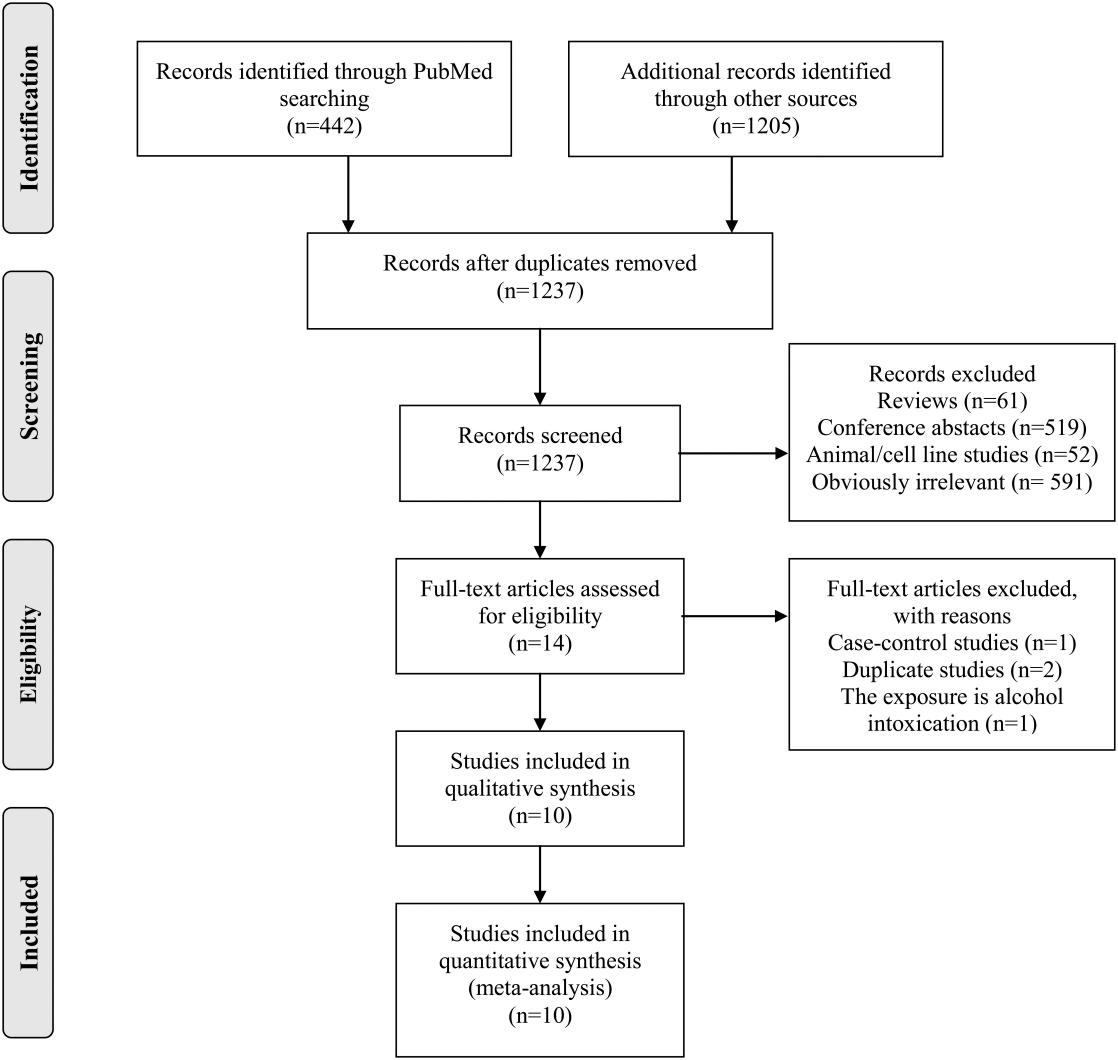
Detailed process of literature search and study selection.

The detailed process of study searching and selection is outlined in Figure 1. We identified 1,237 potentially eligible studies. Most of them were excluded after screening the titles and/or abstracts. Fourteen studies were further evaluated by full-text reading. Finally, 10 prospective studies (10–15, 24–27) were included in our meta-analysis.

All of the included studies were published in English. A total of 441,128 individuals with 10,221 VTE cases were included in these 10 studies. Six studies were from North America and four from Europe. Articles were published between 1996 and 2019. The ascertainment of alcohol intake was based on self-administrated questionnaires (eight studies) or interview (two studies). The quality of the included studies was assessed by NOS, which was summarized in Supplementary Table S2, and the mean NOS score was 7.5 (range, 6–9). Detailed study characteristics are presented in Table 1.

**Table 1:** Main characteristic of the included studies.

**References**	**Country**	**Sex**	**No. of individuals**	**No. of cases**	**Age (years)**	**Exposure assessment**	**Follow-up (years)**	**Categories of alcohol consumption**	**Study name**	**Adjusted variables**
Johansson et al. (25)	Norway	Women	54,632	944	30–60	Questionnaire	13.9 (mean)	≥2.13 standard drinks/week vs. <0.09 standard drinks/week	VEINS	Age, BMI, hypertension, smoking, education, and cancer
Johansson et al. (25)	Norway	Men	53,393	1,110	30–61	Questionnaire	13.10 (mean)	≥4.84 standard drinks/week vs. <0.94 standard drinks/week	VEINS	Age, BMI, hypertension, smoking, education, and cancer
Gaborit et al. (12)	Denmark	Women	28,169	251	50–64	Questionnaire	10.2 (median)	≥21 standard drinks/week vs. 0.1–3.9 standard drinks/week	Danish Diet, Cancer, and Health Study	Age, BMI, smoking, and women's use of HRT, physical activity, education, years in school, diet
Gaborit et al. (12)	Denmark	Men	26,365	352	50–64	Questionnaire	10.2 (median)	≥21 standard drinks/week vs. 0.1–3.9 standard drinks/week	Danish Diet, Cancer, and Health Study	Age, BMI, smoking, physical activity, education, years in school, diet
Varraso et al. (15)	United States	Women	80,263	1,540	30–55	Questionnaire	1984–2008	NR	NHS	Age, physical activity, BMI, total caloric intake, smoking, race, spouse's educational attainment, parity, menopausal status, NSAIDs use, warfarin use, multivitamin supplement use, hypertension, CHD, and rheumatologic disease
Varraso et al. (15)	United States	Men	49,238	1,352	40–75	Questionnaire	1986–2008	NR	HPFS	Age, physical activity, BMI, total caloric intake, smoking, race, NSAIDs use, warfarin use, multivitamin supplement use, hypertension, CHD, and rheumatologic disease
Wattanakit et al. (27)	United States	Both	15,340	468	45–64	Interview	15.5 (mean)	100 vs. 0 g/week	ARIC	Age, race, ARIC field center, sex, and BMI
Hansen-Krone et al. (13)	Norway	Both	26,662	460	25–97	Questionnaire	12.5 (median)	≥7 vs. <1 U/week	The Tromsø Study	Age, sex, BMI, smoking, diabetes, cancer, history of CVD, education, and physical activity
Holst et al. (14)	Denmark	Women	10,153	524	≥20	Questionnaire	19.5 (median)	NR	Copenhagen City Heart Study	Age and calendar time
Holst et al. (14)	Denmark	Men	8,801	445	≥20	Questionnaire	19.5 (median)	NR	Copenhagen City Heart Study	Age and calendar time
Lindqvist et al. (10)	Sweden	Women	24,098	312	25–64	Questionnaire	11 (mean)	≥15 g/day vs. No.	MISS study	Age, cancer, parity, smoking, oral contraceptives use, present regular exercise, and BMI
Lutsey et al. (11)	United States	Women	37,393	1,950	55–69	Questionnaire	19 (median)	≥7 servings/week vs. 0 servings/week	IWHS	Age, kcal, education, smoking, and physical activity
Glynn and Rosner (24)	United States	Men	18,662	358	40–84	Questionnaire	20.1 (median)	NR	Physicians' Health Study	NR
Pahor et al. (26)	United States	Both	7,959	155	≥68	Interview	48,038 person-years	≥1 ounce/day vs. none	EPESE	Age, gender, physical disability, BMI, heart attack, number of hospital admissions in past year, and use of coumarin, diuretics, and corticosteroids

*No., number; BMI, body mass index; NR, not reported; NSAIDs, nonsteroidal anti-inflammatory drugs; CHD, coronary heart disease; CVD, cardiovascular disease; VEINS, Venous thromboEmbolism In Northern Sweden; NHS, Nurses' Health Study; HPFS, Health Professionals Follow-Up Study; ARIC, Atherosclerosis Risk in Communities; MISS, Melanoma Inquiry of Southern Sweden; IWHS, Iowa Women's Health Study; EPESE, Established Populations for Epidemiologic Studies of the Elderly*.

### Highest vs. Lowest Analysis

**Figure 2:**
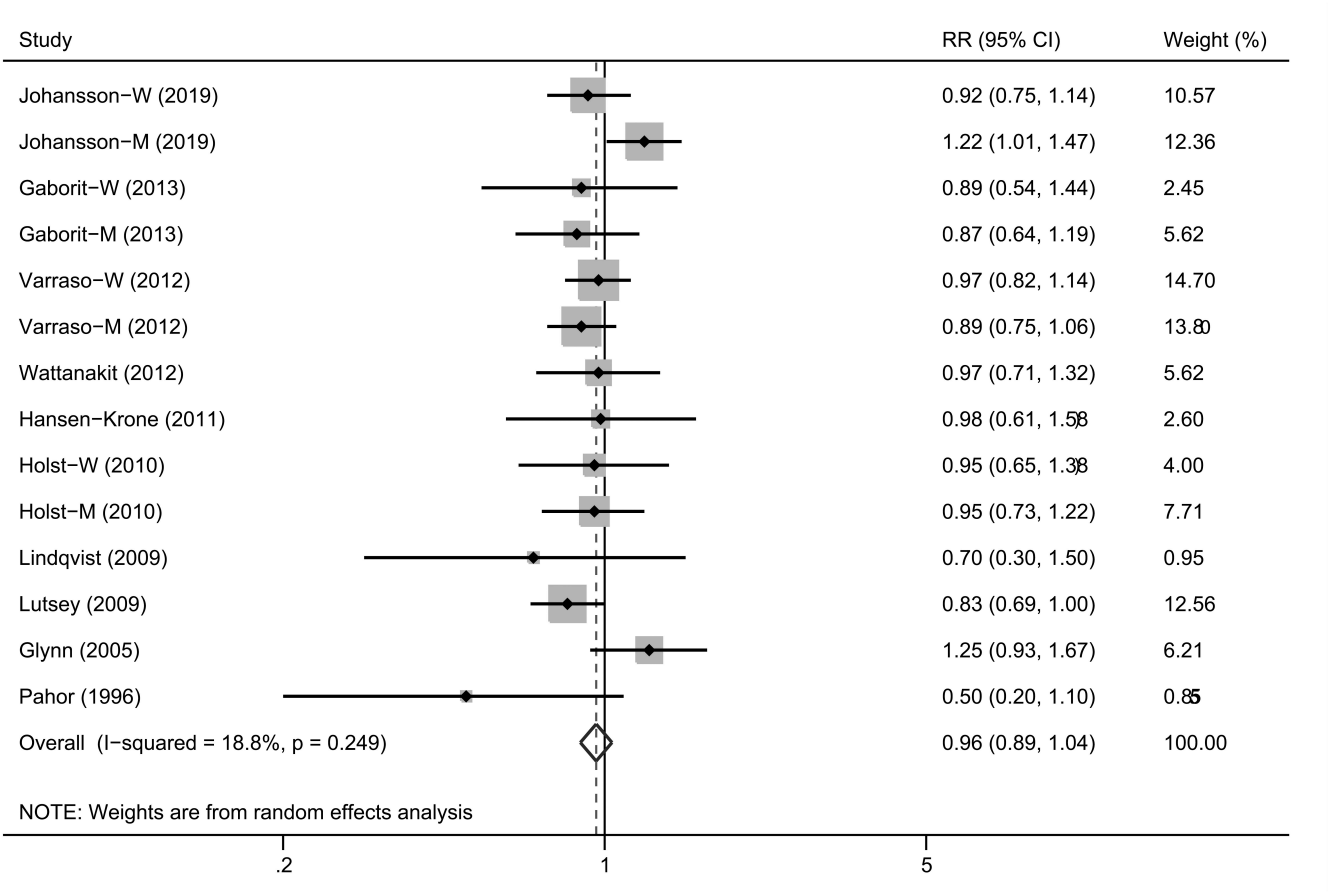
A forest plot showing risk estimates estimating the association between alcohol consumption and venous thromboembolism risk.

Four studies (12, 14, 15, 25) reported the results separately by gender and thus were regarded as independent cohorts. Therefore, 14 cohorts were included in the final summary analysis. Overall, the highest consumption of alcohol was not associated with the VTE risk compared with the lowest group [Figure 2; RR, 0.96 (95% CI, 0.89–1.04), *P* = 0.293]. No obvious heterogeneity of RRs was observed across these studies (*P* = 0.249 for heterogeneity, *I*^2^ = 18.8%).

**Figure 3:**
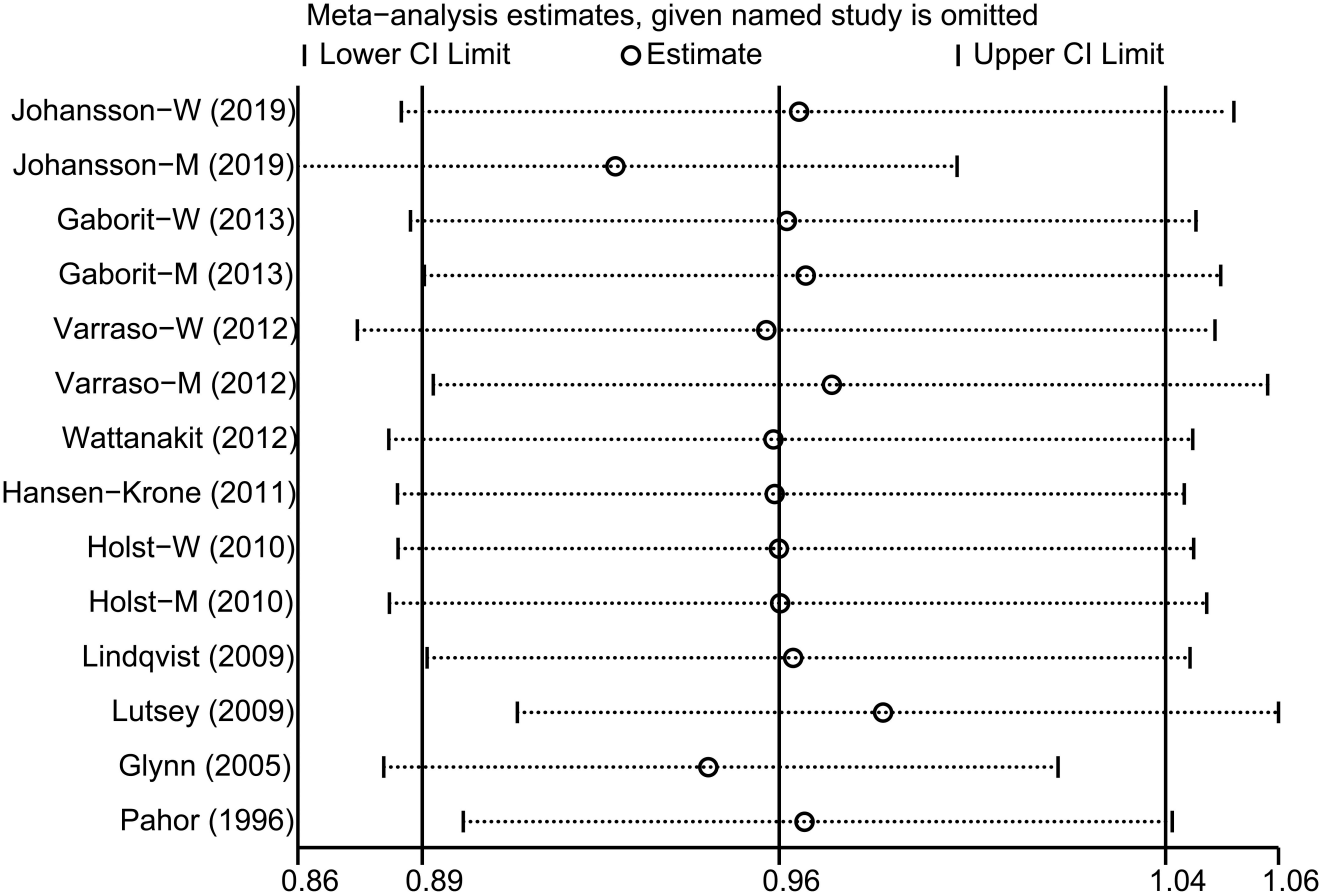
Sensitivity analysis was performed by removing each study in turn and repeating the pooled relative risk estimates.

**Figure 4:**
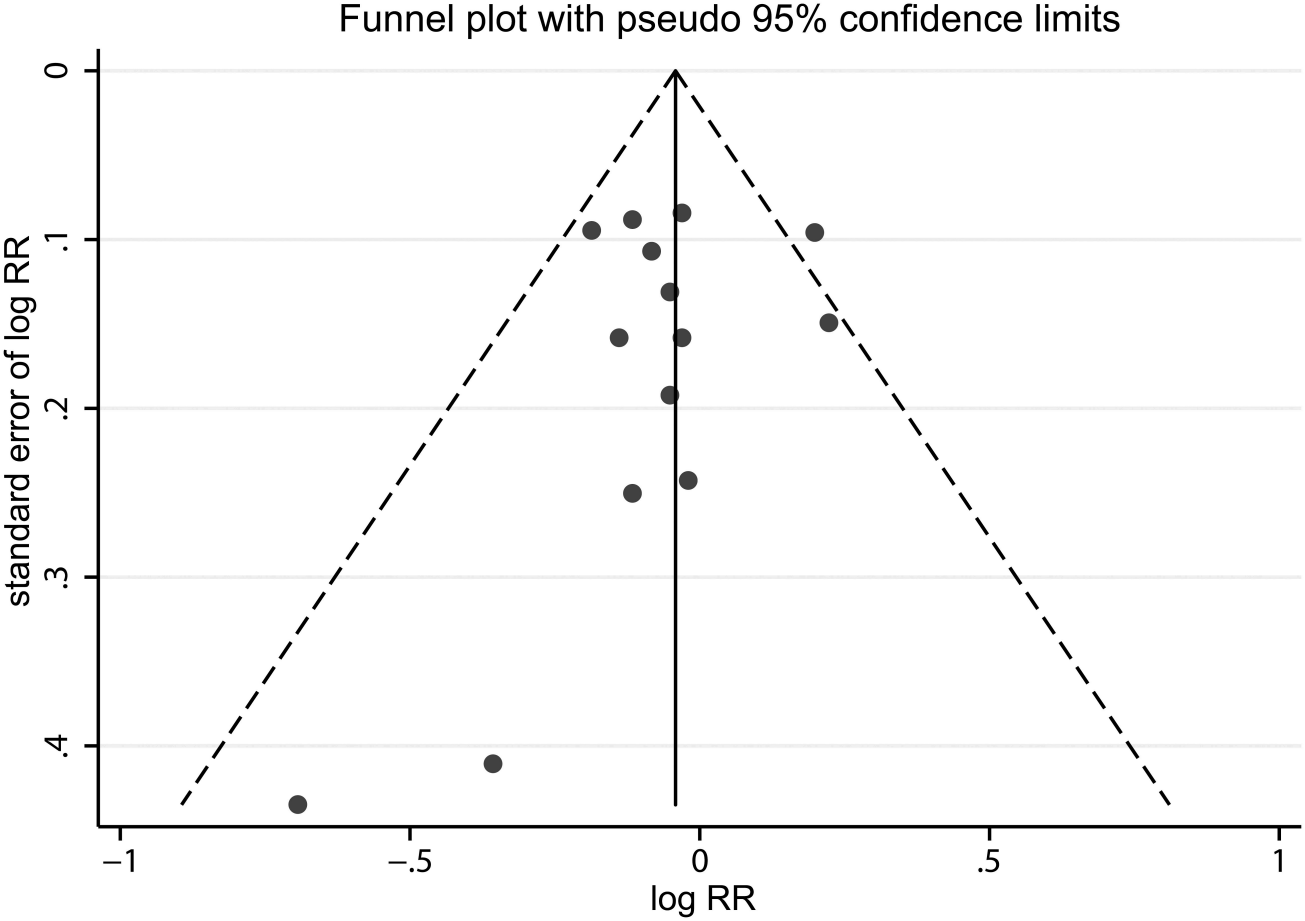
A funnel plot of studies assessing incident venous thromboembolism in highest alcohol consumers compared with the lowest alcohol consumers.

The impact of each included study on the summary RR was determined by repeating the meta-analysis after omitting each study in turn. The results indicated that the pooled risk estimate became statistically significant when the study by Johansson et al. (25) was removed [Figure 3; RR, 0.92 (95% CI, 0.86–0.99)]. Funnel plot (Figure 4) and Egger's and Begg's tests all showed no evidence of publication bias (Egger, *P* = 0.443; Begg, *P* = 0.730). Finally, various stratified analyses were performed. Although no significant association was observed in any subgroups, a borderline significant association between alcohol consumption and VTE risk was observed in women [RR, 0.91 (95% CI, 0.82–1.00)] (Table 2).

**Table 2:** Stratified analysis of the association between alcohol intake and risk for venous thromboembolism.

**Subgroup**	**Included studies**	**Pooled RR (95% CI)**	***P***	**Heterogeneity**		
				***Q***	***I^**2**^* (%)**	***P***
Total	14	0.96 (0.89–1.04)	0.293	16.00	18.8	0.249
**Geographical region**						
Europe	8	0.97 (0.84–1.13)	0.701	14.43	51.5	0.044
North America	6	0.93 (0.85–1.03)	0.178	1.08	0.0	0.956
**Gender**						
Male	5	1.02 (0.88–1.20)	0.766	9.04	55.8	0.060
Female	6	0.91 (0.82–1.00)	0.056	2.00	0.0	0.850
**Number of cases**						
>500	6	0.96 (0.85–1.07)	0.442	9.53	47.5	0.090
≤500	8	0.97 (0.85–1.10)	0.607	6.45	0.0	0.488
**Publication year**						
>2010	8	0.98 (0.90–1.06)	0.575	7.50	6.7	0.379
≤2010	6	0.93 (0.78–1.10)	0.399	7.89	36.6	0.162

*RR, relative risk; CI, confidence interval*.

### Dose–Response Analysis

**Figure 5:**
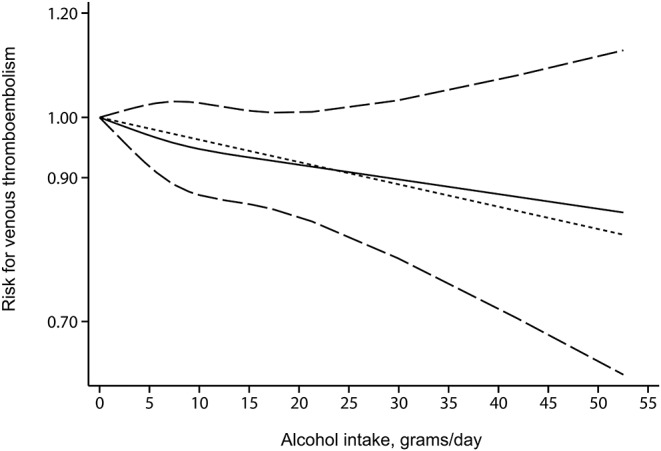
The dose–response association between alcohol intake (grams per day) and venous thromboembolism risk. The solid line and the long dash line represent the estimated risk estimates and their 95% CIs. Short dash line represents the linear relationship.

Seven studies (nine cohorts) (10–13, 25–27) were available for the dose–response analysis. Alcohol intake for the different categories, together with RRs and CIs, was provided in Supplementary Table S3. After evaluating dose–response patterns for alcohol intake per day, we observed a linear decrease in VTE risk with increasing alcohol intake (*P* = 0.634 for nonlinearity). However, the reduced risk was not statistically significant (Figure 5).

## Discussion

The present meta-analysis, involving ~400 thousand participants and more than 10 thousand patients with VTE from 10 prospective studies (14 cohorts), indicated that alcohol intake was not associated with the risk of VTE overall. However, a borderline significant association between alcohol consumption and VTE risk was observed in women. Sensitivity analysis indicated that the pooled risk estimate became statistically significant when the study by Johansson et al. (25), based on men, was removed. These results implied a potential protective role of alcohol (in moderation) against VTE in women. To the best of our knowledge, this is the first meta-analysis aimed to investigate the association between alcohol consumption and VTE risk.

Emerging studies have indicated that lack of physical activity (7), smoking (8), and obesity (28) were the potential risk factors for incident VTE, whereas controversial results were reported for alcohol intake (12, 15, 24, 27). Several mechanisms have been proposed to support a potential relationship between alcohol intake and VTE risk. Dimmitt et al. (29) reported that alcohol intake was associated with factor VII, tissue plasminogen activator (tPA), and plasminogen activator inhibitor-1 (PAI-1). Mukamal et al. (30) found that light-to-moderate alcohol intake was related with lower levels of coagulatory factors, but higher consumption was related with impaired fibrinolytic potential, which implied a J- or U-shaped relationship between alcohol intake and hemostatic parameters. However, we failed to find a U-shaped association in the dose–response analysis (*P* = 0.634 for non-linearity).

Risk differences may exist between the genders for the association between alcohol intake and VTE risk. Johansson et al. (25) found that high alcohol consumption was associated with an increased risk of VTE in men but not in women. In contrast, several studies (10, 11, 31) have suggested an inverse relationship between alcohol intake and VTE risk in women. In our study, we also performed subgroup analysis by gender. As a result, a borderline significant association between alcohol consumption and VTE risk was observed in women. However, due to the limited number of eligible studies in this stratified analysis, we may have limited power to detect possible associations in these subgroups. In addition, we were not able to investigate the dose–response relation in women, as most included studies did not report data separately by sex.

Our meta-analysis has several strengths. First, only prospective cohort studies were included in this meta-analysis, which avoided the selection and recall bias in case–control studies. Second, the total sample size was large, and no obvious heterogeneity was observed across studies, which enhanced the robustness of the findings. Third, various analyses were performed in this study, including sensitivity analysis, dose–response analysis, subgroup analyses, and publication bias analysis.

This meta-analysis also has several limitations. First, like all meta-analyses, our study has the limitation of being a retrospective analysis. Second, various cut-off values for the categories of alcohol intake were used across studies, which led to a certain degree of heterogeneity. Third, the inadequate adjustment of all known confounding factors in some included studies may distort the summary risk estimate (32). Fourth, the results of our study are likely to be only generalizable to Western populations and may not be generalizable to Asian populations. Fifth, some studies included former drinkers in the reference group, which may distort the association. If alcohol consumption has a protective role in VTE, the inclusion of former drinkers in the reference group can result in an underestimate of the true association. Finally, because of the limited data, a subgroup analysis for pulmonary embolism, unprovoked, provoked VTE, and beverage type was not performed.

In conclusion, the results from this meta-analysis suggest that alcohol intake is not related with the risk of VTE. Further large well-designed cohort studies are warranted to investigate a potential protective role of alcohol against VTE in women.

## Data Availability Statement

The datasets generated for this study are available on request to the corresponding author.

## Author Contributions

MC and MJ originated and designed the study and drafted the manuscript. TC and XH searched the databases. MC and YJ contributed to the data extraction and data analyses. MJ and TC assisted with literature selection. TC reviewed and edited the manuscript. All authors have read and approved the final manuscript.

### Conflict of Interest

The authors declare that the research was conducted in the absence of any commercial or financial relationships that could be construed as a potential conflict of interest.
