# Supplementary material for: Alcohol Consumption and Risk for Venous Thromboembolism: A Meta-Analysis of Prospective Studies
Source: Front Nutr. 2020 Apr 2;7:32. doi: 10.3389/fnut.2020.00032 (PMC7145405; doi:10.3389/fnut.2020.00032)
Supplement: Supplementary file 1 [file Data_Sheet_1.docx]

**Supplementary Text S1 The search strategy in PubMed.**

#1 venous thromboembolism [MeSH]

#2 VTE [tiab] OR thromboembolism [tiab] OR venous thrombosis [tiab] OR pulmonary embolism [tiab]

#3 #1 OR #2

#4 ethanol [MeSH]

#5 alcohol* [tiab] OR alcoholic [tiab] OR ethanol [tiab] OR wine [tiab] OR beer [tiab] OR liquor [tiab]

#6 #4 OR #5

#7 cohort [tiab] OR nested case-control [tiab] OR case-cohort [tiab] OR trial [tiab]

#8 #3 and #6 and #7
