# Supplementary material for: Alcohol Consumption and Risk for Venous Thromboembolism: A Meta-Analysis of Prospective Studies
Source: Front Nutr. 2020 Apr 2;7:32. doi: 10.3389/fnut.2020.00032 (PMC7145405; doi:10.3389/fnut.2020.00032)
Supplement: Supplementary file 2 [file Table_1.docx]

| **Supplementary Table S1 PICOS criteria for inclusion and exclusion of studies** | |
| --- | --- |
| **Parameter** | **Inclusion criteria** |
| Population | Individuals without venous thromboembolism at baseline |
| Intervention/  exposures | Alcohol consumption |
| Comparison | Dose-response association |
| Outcomes | Venous thromboembolism |
| Type of study | Nested case-control, cohort and case-cohort studies, and follow-up studies of randomized clinical trials |
