# Supplementary material for: Alcohol Consumption and Risk for Venous Thromboembolism: A Meta-Analysis of Prospective Studies
Source: Front Nutr. 2020 Apr 2;7:32. doi: 10.3389/fnut.2020.00032 (PMC7145405; doi:10.3389/fnut.2020.00032)
Supplement: Supplementary file 3 [file Table_2.docx]

| **Supplementary Table S2** **Methodological quality of studies included in the meta-analysis** | | | | | | | | | |
| --- | --- | --- | --- | --- | --- | --- | --- | --- | --- |
| First author, year | Representativeness of the exposed  cohort | Selection of the  unexposed  cohort | Ascertainment  of exposure | Outcome of  interest not  present at  start of study | Comparability ^a^ | Outcome  assessment | Follow-up long enough for outcomes to occur ^b^ | Adequacy  of follow-up  of cohorts | Total  quality  scores |
| Johansson et al., 2019 | ☆ | ☆ | - | ☆ | ☆☆ | ☆ | ☆ | ☆ | 8 |
| Gaborit et al., 2013 | ☆ | ☆ | - | ☆ | ☆☆ | ☆ | ☆ | ☆ | 8 |
| Varraso et al., 2012 | ☆ | ☆ | - | ☆ | ☆☆ | ☆ | ☆ | ☆ | 8 |
| Wattanakit et al., 2012 | ☆ | ☆ | ☆ | ☆ | ☆☆ | ☆ | ☆ | ☆ | 9 |
| Hansen-Krone et al., 2011 | ☆ | ☆ | - | ☆ | ☆☆ | ☆ | ☆ | - | 7 |
| Holst et al., 2010 | ☆ | ☆ | - | ☆ | ☆ | ☆ | ☆ | ☆ | 7 |
| Lindqvist et al., 2009 | ☆ | ☆ | - | ☆ | ☆☆ | ☆ | ☆ | - | 7 |
| Lutsey et al., 2009 | ☆ | ☆ | - | ☆ | ☆☆ | ☆ | ☆ | ☆ | 8 |
| Glynn et al., 2005 | ☆ | ☆ | - | ☆ | - | ☆ | ☆ | ☆ | 6 |
| Pahor et al., 1996 | ☆ | ☆ | ☆ | ☆ | ☆☆ | ☆ | - | - | 7 |
| ^a^ A maximum of two stars can be awarded for this item. Studies adjusting for age received one star while studies additionally adjusting for other confounders received an additional star.  ^b^ A cohort study with more than 10 years of follow-up was assigned one star. | | | | | | | | | |
