# Supplementary material for: Alcohol Consumption and Risk for Venous Thromboembolism: A Meta-Analysis of Prospective Studies
Source: Front Nutr. 2020 Apr 2;7:32. doi: 10.3389/fnut.2020.00032 (PMC7145405; doi:10.3389/fnut.2020.00032)
Supplement: Supplementary file 4 [file Table_3.docx]

| **Supplementary Table S3 Alcohol intake for the different categories used for the dose-response analysis, together with relative risks (RRs) and confidence levels (CIs).** | | | | |
| --- | --- | --- | --- | --- |
| First author | Alcohol intake (g/d) | RR | LL | UL |
| Johansson (men) | 0.81 | 1 | 1 | 1 |
|  | 5.69 | 1.05 | 0.87 | 1.27 |
|  | 9.03 | 1.18 | 0.98 | 1.42 |
|  | 10 | 1.22 | 1.01 | 1.47 |
| Johansson (women) | 0.08 | 1 | 1 | 1 |
|  | 1.09 | 0.83 | 0.68 | 1.00 |
|  | 2.76 | 0.92 | 0.75 | 1.13 |
|  | 4.54 | 0.92 | 0.75 | 1.14 |
| Gaborit (women) | 3.43 | 1 | 1 | 1 |
|  | 15.34 | 0.94 | 0.71 | 1.25 |
|  | 29.91 | 1.01 | 0.64 | 1.60 |
|  | 41.91 | 0.89 | 0.54 | 1.44 |
| Gaborit (men) | 3.43 | 1 | 1 | 1 |
|  | 15.34 | 0.76 | 0.56 | 1.04 |
|  | 29.91 | 0.79 | 0.53 | 1.18 |
|  | 41.91 | 0.87 | 0.64 | 1.19 |
| Wattanakit | 0 | 1 | 1 | 1 |
|  | 7.14 | 0.93 | 0.72 | 1.20 |
|  | 21.29 | 0.97 | 0.71 | 1.32 |
| Hansen-Krone | 0 | 1 | 1 | 1 |
|  | 1.00 | 0.89 | 0.69 | 1.16 |
|  | 3.00 | 1.10 | 0.82 | 1.38 |
|  | 9.00 | 0.98 | 0.70 | 1.39 |
|  | 17.00 | 0.98 | 0.61 | 1.58 |
| Lindqvist | 0 | 1 | 1 | 1 |
|  | 2.50 | 0.90 | 0.60 | 1.30 |
|  | 7.50 | 0.60 | 0.40 | 1.10 |
|  | 12.50 | 0.40 | 0.20 | 0.96 |
|  | 17.50 | 0.70 | 0.30 | 1.50 |
| Pahor | 0 | 1 | 1 | 1 |
|  | 14.18 | 0.60 | 0.40 | 0.90 |
|  | 52.53 | 0.50 | 0.20 | 1.10 |
| Lutsey | 0 | 1 | 1 | 1 |
|  | 3.13 | 1.02 | 0.92 | 1.13 |
|  | 8.93 | 0.87 | 0.71 | 1.06 |
|  | 15.18 | 0.83 | 0.69 | 1.00 |
